# Cognitive Decline and Reorganization of Functional Connectivity in Healthy Aging: The Pivotal Role of the Salience Network in the Prediction of Age and Cognitive Performances

**DOI:** 10.3389/fnagi.2016.00204

**Published:** 2016-08-29

**Authors:** Valentina La Corte, Marco Sperduti, Caroline Malherbe, François Vialatte, Stéphanie Lion, Thierry Gallarda, Catherine Oppenheim, Pascale Piolino

**Affiliations:** ^1^Laboratory of Memory and Cognition, Institute of Psychology, University Paris DescartesParis, France; ^2^INSERM UMR S894, Center of Psychiatry and Neurosciences, University Paris DescartesParis, France; ^3^IDEX ‘Dynamique du Vieillir’, Sorbonne Paris Cité, University Paris DiderotParis, France; ^4^INSERM U894, Center of Psychiatry and Neurosciences, Department of Radiology, University Paris DescartesParis, France; ^5^Department of Computational Neuroscience, University Medical Center EppendorfHamburg, Germany; ^6^Clinic and Polyclinic of Neurology, University Medical Center EppendorfHamburg, Germany; ^7^Brain Plasticity Unit, CNRS U8249, ESPCI ParisTechParis, France; ^8^Laboratory of Physiopathology of Psychiatric Diseases, Center of Psychiatry and NeurosciencesParis, France; ^9^University Institute of France, IUFParis, France

**Keywords:** resting state, rs-fMRI, episodic memory, autobiographical memory, executive functions, functional connectivity, machine learning, aging

## Abstract

Normal aging is related to a decline in specific cognitive processes, in particular in executive functions and memory. In recent years a growing number of studies have focused on changes in brain functional connectivity related to cognitive aging. A common finding is the decreased connectivity within multiple resting state networks, including the default mode network (DMN) and the salience network. In this study, we measured resting state activity using fMRI and explored whether cognitive decline is related to altered functional connectivity. To this end we used a machine learning approach to classify young and old participants from functional connectivity data. The originality of the approach consists in the prediction of the performance and age of the subjects based on functional connectivity by using a machine learning approach. Our findings showed that the connectivity profile between specific networks predicts both the age of the subjects and their cognitive abilities. In particular, we report that the connectivity profiles between the salience and visual networks, and the salience and the anterior part of the DMN, were the features that best predicted the age. Moreover, independently of the age of the subject, connectivity between the salience network and various specific networks (i.e., visual, frontal) predicted episodic memory skills either based on a standard assessment or on an autobiographical memory task, and short-term memory binding. Finally, the connectivity between the salience and the frontal networks predicted inhibition and updating performance, but this link was no longer significant after removing the effect of age. Our findings confirm the crucial role of episodic memory and executive functions in cognitive aging and suggest a pivotal role of the salience network in neural reorganization in aging.

## Introduction

The cognitive and neural changes accompanying healthy aging are a crucial topic in cognitive neuroscience. The age-related cognitive decline has emerged as a major societal concern given the increase in the elderly population. Nevertheless, not all cognitive domains are equally affected by age, and not all cognitive processes show age-related decline. There is compelling evidence that executive functions and memory are the most severely impaired cognitive domains in this population (Salthouse et al., 2003).

Executive functions are seen as high-level cognitive processes responsible for flexible and adaptive behavior (Miyake et al., 2000). Thus, they play a fundamental role in dealing with complex situations in everyday life. Moreover, they largely contribute to the effective functioning of other cognitive processes, such as memory. Notably, some authors have proposed that the central deficit responsible for the general cognitive decline in aging is linked to inefficient executive functioning (West, 1996; Salthouse et al., 2003). At the neural level, this decline may be accounted by the functional and structural reorganization of the frontal lobes with aging (Moscovitch and Winocur, 1995; Cabeza, 2002; Grady et al., 2005; Fjell and Walhovd, 2010).

The cognitive domain that has received the greatest attention in normal aging is memory. Many older adults complain of increased memory lapses as they age and a major focus of research has been trying to distinguish memory decline attributable to normal aging from that related to pathological aging, in particular in Alzheimer’s disease.

Within the framework of long-term memory, dissociation between spared semantic memory (i.e., general knowledge about the world, words and concept) and impaired episodic memory (i.e., memory for personally experienced events that occurred in a particular place at a specific time) has been reported in aging.

The episodic memory decline in older adults may result from a parallel impairment of strategic control processes involved in encoding and memory retrieval. Accordingly, several studies using laboratory tests of episodic memory have highlighted a reduction in the use of effortful encoding strategies, which are mainly related to prefrontal brain regions (Hara and Naveh-Benjamin, 2015). In the same line, considerable evidence points to deficits in effortful retrieval in older adults. In particular several studies have shown impaired free recall along with normal cued recall or recognition (Ward and Maylor, 2005).

These findings show that memory decline in cognitive aging is strongly related to executive functions.

Moreover, a large number of studies have investigated cognitive aging changes in episodic performance via autobiographical memory, which is defined as the memory for personal experiences that underlies the personal identity and the temporal continuity of an individual (Conway and Pleydell-Pearce, 2000). A distinction between an episodic and a semantic component has also been proposed in this domain. The former refers to memory for personal events situated in a specific spatiotemporal context, while the latter refers to general knowledge about one’s own past and about oneself. Again, dissociation between spared semantic and impaired episodic autobiographical memory has been documented in the elderly (Levine et al., 2002; Piolino et al., 2002, 2006; St. Jacques and Levine, 2007; Martinelli et al., 2013a). The deficit of the episodic component of autobiographical memory has been linked to a reduced availability of executive resources (Conway and Pleydell-Pearce, 2000; Conway, 2005; Piolino et al., 2010; Coste et al., 2011), and to a reduced recruitment of the underlying brain structures (Martinelli et al., 2013b).

Functional magnetic imaging (fMRI) has been widely used in order to link age-related cognitive decline with patterns of altered brain function. A consistent finding in the fMRI literature is that healthy old adults present higher brain activation in a wide range of cognitive tasks (Cabeza, 2002). On the other hand some studies have highlighted a reduced brain activity in cognitive aging (Damoiseaux et al., 2008). More recently, an increasing number of investigations have focused on the study of the relationship between cognitive functions and functional connectivity mainly derived from resting state fMRI (rs-fMRI). rs-fMRI is the study of the interregional correlation of spontaneous fluctuation in brain activity while subjects are not engaged in any specific cognitive task. It represents a promising candidate for studying the complex neural organization underlying cognition and its modification due to different conditions (normal aging, psychiatric and neurodegenerative disorders) without task-specific confounds.

The use of rs-fMRI to study functional connectivity has allowed the identification of a set of networks named resting state networks (RSNs). These networks are commonly identified across young healthy subjects (Damoiseaux et al., 2006) and have shown high reproducibility (Guo et al., 2012).

The most widely studied RSN is the default mode network (DMN), composed of regions that are deactivated during the performance of goal-directed tasks and that show high levels of activity at rest. Buckner et al. (2008) defined the core regions associated with the brain’s default network: the ventral/dorsal medial prefrontal cortex (PFC), the posterior cingulate and retrosplenial cortex, the inferior parietal lobule and the hippocampal formation (including the entorhinal cortex and parahippocampal cortex).

Beside the DMN, other networks of intrinsic brain connectivity have been described in healthy populations. These findings indicate that the human brain has a network-based organization even at rest. In recent years, a consistent number of investigations have focused on the salience network (Menon, 2015; Metzler-Baddeley et al., 2016). The salience network is an intrinsically connected large-scale network anchored in the anterior insula and the dorsal anterior cingulate cortex. With the anterior insula as its dynamic hub, the salience network contributes to a variety of complex brain functions through the integration of sensory, emotional and cognitive information (Menon, 2015).

Recently, a direct link between inter-individual variability in functional connectivity measured at rest in specific networks and cognitive functions has been documented. For example, Cole et al. (2012) reported that the global connectivity of the lateral prefrontal cortex (LPFC) predicted individual differences in fluid intelligence. A correlation between the strength of the connectivity between the two major nodes of the DMN, the ventral medial prefrontal cortex (vMPFC) and the posterior cingulate cortex (PCC), and working memory abilities (Hampson et al., 2006), or episodic memory performances (Tambini et al., 2010) has been reported.

This approach has proved fruitful in describing the neural reorganization in aging. Several studies have reported reduced connectivity between the two major nodes of the DMN, the vMPFC and the PCC (Andrews-Hanna et al., 2007; Damoiseaux et al., 2008; Balsters et al., 2013; Mevel et al., 2013). Other networks with reduced connectivity are the fronto-parietal attentional (Andrews-Hanna et al., 2007; Balsters et al., 2013; Vergun et al., 2013), the sensorimotor (Meier et al., 2012) and the salience networks (Meier et al., 2012; Onoda et al., 2012). In particular, the connectivity profile in the salience network has been shown to be the best feature to classify young and old participants using a machine learning approach (Meier et al., 2012), and that internetwork connectivity between the salience and the visual and the auditory networks is reduced in aging (Onoda et al., 2012).

Moreover, a direct link between reduced network connectivity and impaired cognitive functions has been reported in aging. In particular, decreased connectivity between the anterior and the posterior node of the DMN correlated with a composite measure of memory (Andrews-Hanna et al., 2007). Concerning autobiographical memory, a correlation has been reported between the strength of connectivity between the posterior node of the DMN and middle temporal structures, comprising the hippocampus, and an episodic autobiographical fluency, and between semantic autobiographical fluency and the connectivity between the anterior node of the DMN and the ventral anterior cingulate cortex (Mevel et al., 2013). Additionally, reduced connectivity within the DMN and the salience network has been related to a decline in executive functions in aging (Damoiseaux et al., 2008; Onoda et al., 2012). Taken together, these findings highlight the pertinence of using rs-fMRI to explain the complex neuronal reorganization linked to the cognitive decline observed in aging (Andrews-Hanna et al., 2007; Damoiseaux et al., 2008; Onoda et al., 2012; Sala-Llonch et al., 2015).

The principal aim of this study was to further characterize the brain functional reorganization related to cognitive aging in order to shed light on the network reorganization related to cognitive decline in older adults, in particular linked to episodic memory and executive functions.

The originality of the study consisted in using a machine-learning approach to predict age and cognitive performance from functional connectivity patterns. Gottlieb (2012) recently proposed that a closer integration of machine learning in cognitive neuroscience has the potential to answer fundamental questions about cognitive functions. Such an approach has already proven its validity in recent investigations of neuropsychological features in neurology or psychiatry (Costafreda et al., 2011; Quintana et al., 2012). Developed from a connectionist approach, this modeling strategy has several advantages over computationalist methods: it can be easily applied to multi-modal data analysis, and in addition it is not constrained by *a priori* assumptions or abstractions on the data. The model is built using the input feature vectors (e.g., multimodal recordings of cognitive tasks) and matching this vector with expected outputs (e.g., prediction of cognitive variables). Once the model has been built, it is then confronted to a new independent test dataset to estimate its validity.

Therefore, we used multivariate statistical techniques to classify young and old participants using a machine learning approach (Meier et al., 2012). We hypothesized that aging would disrupt not only DMN but also the salience network (Onoda et al., 2012) and that this pattern of modifications at the functional level would be related to cognitive changes in particular in episodic memory and executive functions.

## Materials and Methods

### Subjects

Twenty-seven healthy participants, 17 young adults (YA: nine females, mean age 28.75 ± 4.62) and 10 old adults (OA: four females, mean age 70 ± 5.01) took part in the study. These participants represent a subgroup of an fMRI activation study whose data have already been published elsewhere (Martinelli et al., 2013b). All participants gave their informed written consent and the study was approved by the local ethics committee of Sainte Anne Hospital (CPP Ile de France 3 n°2687). All subjects were right-handed (according to the Edinburgh Handedness Inventory; Oldfield, 1971), and native French speakers. Medical, demographic, and psychometric data were obtained prior to the scanning session. All participants were unmedicated, living at home and rigorously screened for uncontrolled hypertension and cerebrovascular risk factors. Exclusion criteria included presence of a history of alcohol or substance abuse, head trauma, major disease affecting brain function, neuropsychiatric disorders (tested with the Mini-International Neuropsychiatric Interview, Sheehan et al., 1998), depression [tested with the Geriatric Depression Scale, Yesavage et al., 1983, cut-off score > 10; YA: 2.65 ± 2.67; OA: 3.4 ± 2.91; student *t*-test: *t*(25) = 0.68, *p* = 0.5], abnormal general cognitive functioning as assessed by the Mattis scale (Mattis, 1976, cut-off score < 136; young adults: 142.50 ± 1.26 and old adults: 139.90 ± 3.04). The two groups were matched according to their verbal abilities and crystallized intelligence as assessed by the Mill Hill test [Deltour, 1993; percentile score for YA: 54.38 ± 26.83 and OA: 53.83 ± 30.82, student *t*-test: *t*(25) = 0.04, *p* = 0.97].

### Procedure

The whole experimental session comprised three phases (pre-scanning, scanning, post-scanning).

During the first phase (pre-scanning interview), participants were tested for exclusion and inclusion criteria, they underwent a medical examination, neuropsychological assessment and completed the Taste and Interest Questionnaire (TIQ) that was employed to create personal cues used for the autobiographical memory tasks during the scanning and post-scanning sessions. During the scanning session, participants were first trained in the autobiographical task outside the scanner, and then a high-resolution 3D structural image was acquired as well as a resting state functional session. Subsequently they participated in an activation protocol during which they performed the autobiographical task from personal cues (other than those used for training). After the fMRI protocol, during the post-scanning session (debriefing) subjects were asked to re-evoke their autobiographical memories from the same cues seen under the scan. Here we will mainly focus on the measures of neuropsychological assessment and autobiographical memories at debriefing and the rs-fMRI (for details on the activation protocol results see Martinelli et al., 2013c).

### Behavioral Measures

#### Autobiographical Memory

In the pre-scanning interview, exclusion and inclusion criteria were verified by means of a clinical assessment and psychometric tests, and then neuropsychological tests and the TIQ were submitted to subjects. The aim of the TIQ was mainly to collect information so as to create personalized specific event cues for each participant. Twenty-four activities or interests for episodic autobiographical memory (EAM) were selected from the TIQ (for a complete description of personal cue elaboration see Martinelli et al., 2013b; Sperduti et al., 2013).

The participants were first invited to take part in a training session before the fMRI scanning. Participants received detailed explanations on the nature of the task and participated in a brief simulation of the experiment on a laptop. For the two EAM tasks (mental retrieval under the scanner and aloud retrieval at debriefing) we gave the following explanations:

- EAM was defined as a memory of a single event that occurred at a specific time and place, of short duration, lasting less than 24 h. Participants were instructed to mentally relive personal episodes prompted by cues and to try to retrieve spatiotemporal, affective and perceptual details (such as time, location, perceptions, feelings, scenery, and people present in the scene) (e.g., “a unique memory linked to a trip to New York”).

After the scanning session, in order to score the memories retrieved in the scanner, participants were asked to recall each memory again. EAMs were rated for richness and specificity on standard scales (Levine et al., 2002; Piolino et al., 2009; Martinelli et al., 2013a). More precisely, the presence of a sense of remembering with recall of specific spatial and temporal details, and other contextual and phenomenological details in each evocation was noted (1 point per type of detail, maximum 4; e.g., “I remembered my visit to the Palace of Tokyo in Paris, in August 2009, as if I was still there. I was with Chiara in a room at the exhibition on the first floor in the dark to see the TV reports and talk with other visitors, it was 6 pm and still very warm, but it was worth it!, after that we went to the restaurant of the outdoor museum on the bank of the Seine...”). For each participant we computed a global ratio of specificity **(EAM** score) totaling up the sum of spatiotemporal, other contextual and phenomenological details divided by the sum depending on the number of recalls.

#### Episodic Memory

The Free and Cued Selective Reminding test (FCRT) was used to assess episodic memory capacities (Grober and Buschke, 1987; French version Van der Linden et al., 2004).

Different studies have shown the validity of this tool to discriminate healthy old adults from prodromal Alzheimer’s disease (AD) patients (Lemos et al., 2015; Papp et al., 2015).

The test begins with a study phase designed to control attention and cognitive processing to identify memory impairment that it is not secondary to other cognitive deficits. During the encoding phase, subjects have to identify words in response to category cues (fruits, clothing, etc.). In the test phase, subjects are asked to recall the items they learned (free recall). The category cues are used to prompt recall of items not retrieved by free recall to generate a score called cued recall. We calculated the sum of free and cued recall termed **EPI total recall**.

#### Executive Functions

For the assessment of the executive functions, we used the Trail Making Test (TMT; Reitan, 1958) and verbal fluency (Cardebat et al., 1990) as a measure of behavioral and cognitive flexibility respectively. For the TMT we computed the difference in execution time between part B and part A (**TMTB-A** score). Concerning the verbal fluency we added the total number of words for the lexical (number of words starting with the letter P) and the semantic (number of words belonging to the semantic category “animals”) fluency (**FLU** score).

As a measure of inhibition we used the Victoria STROOP test (Stroop, 1935). In particular we computed the difference between the time of denomination of the interference part and the denomination part (**INHIB** score). For up-dating (**UP-D** score) in working memory we used the running span (Quinette et al., 2003). For visuo-spatial working memory we used a battery assessing the visuo-spatial span (**VSS** score) forward and backward task (sum of the two spans), and the short-term binding (**STB** score) ability using a visuo-spatial binding task (Picard et al., 2012).

### fMRI Data Acquisition

All data were acquired with a 3 T scanner (Discovery MR 750, General Electric Healthcare). The anatomical scan used an inversion recovery 3-D T1-weighted gradient-echo sequence of images (TE = 4.3 ms, TR = 11.2 ms, TI = 400 ms, matrix = 384 × 384, slice thickness = 1.2 mm). Functional resting state images were acquired using a gradient echo echoplanar (EPI) sequence (TE = 30 ms, TR = 2000 ms, flip angle = 90°, matrix = 64 × 64, slice thickness = 3 mm, 42 contiguous sections). The functional scan lasted 5 min.

### fMRI Data Analysis

#### Extraction of Networks and Regions of Interest

We extracted resting state networks from an independent set of resting state data available on the 1000 Functional Connectome Project^1^. This dataset contains functional scans of 86 subjects (45 females, age 19–85 years) acquired with a 3T scanner with the following parameters: TR = 2 s, 23 slices, acquisition type = sequential ascending.

All data were processed using SPM5 software (Statistical Parametric Mapping 5, Welcome Department of Cognitive Neurology, UK^2^). Standard pre-processing procedures were applied to functional data. EPI volumes were corrected for slice timing, subject’s rigid motion and spatially smoothed using an isotropic Gaussian kernel filter of 5 mm full-width half-maximum.

After preprocessing, resting state networks were extracted using group spatial independent component analysis (sICA) as implemented in the Network Detection using ICA (NEDICA) software (Perlbarg et al., 2008). Networks were first extracted for each subject in her/his native space. The spatial ICs obtained for each subject were then normalized in the MNI standard space and clustered into classes representative of the population. To do so we used a hierarchical clustering algorithm (Hartigan, 1975). All normalized spatial maps in each class were then averaged and thresholded at *p* < 0.05 using *t*-test statistics corrected for multiple comparisons using a false discovery rate (FDR) approach (Genovese et al., 2002). Threshold maps were visually inspected to select maps exhibiting a known spatial organization. These maps are referred to as functional networks (for a similar procedure, see Malherbe et al., 2014).

Then, regions of interest were extracted from the functional networks obtained by selecting the maxima of connectivity peaks of the group functional networks. All regions of interest were defined as a sphere of 10 voxels in the Montreal Neurological Institute space (voxel size: 3.5 mm × 3.5 mm × 3.5 mm). These regions of interest were then used to extract the time course of our functional resting state data after applying the same preprocessing steps described above. The mean time series were calculated across all voxels within each region of interest in the MNI space, for each subject. The motion parameters, as well as signals from white matter and CSF and linear and quadratic drifts were then used as covariates of no interest in a general linear model for the mean time courses in each region considered in the analysis. Regions were then grouped in networks. For each network, the time course was obtained by meaning all time courses of implicated regions. Finally, a correlation matrix of the average time course between each pair of networks was computed (see Table in Appendix 1). This measure was used in the following steps of data analysis.

### Machine Learning Analyses

In the present study, using a machine learning approach, we performed two analyses: supervised feature selection, and supervised regression. All the analyses were performed after volume correction: all variables were orthogonalized according to the cortical volume.

Feature selection was performed using the orthogonal forward regression (OFR) algorithm (Chen et al., 1989), which was used to select the best network activities to predict either the age of a subject, or one of the cognitive variables. All the descriptors were considered as vectors (one fMRI was considered as one vector), and we analyzed iteratively the best set of features to model one expected output at a time. Given the feature vectors f_i,i∈[1..N] and the output vector Ω, the OFR feature selection approach follows three steps:

(I) All descriptors are ranked according to their distance to the output. The distance is computed as the cosine of the angle 𝜃 between the vector and the output: 𝜃 = cos(f_i,Ω).
(II) The descriptor with the lowest absolute angle (maximum cosine) is ranked first. All remaining descriptors and the output are projected into the null-space of the best descriptor.
(III) The selected descriptor is stored and removed from the set, and the algorithm iterates on the remaining orthogonalized features.

In order to control for the relevance of the selected features, we used a probe variables approach (Stoppiglia et al., 2003). We inserted in the feature set 100 randomly drawn vectors. The rank distribution of these probes indicated the risk of a descriptor containing information that could be explained by chance. We fixed a threshold of 5% of probes in our investigation, and selected only descriptors above that threshold. The variables were analyzed after volumetric correction (the correction was performed by orthogonalizing all variables to the null-space of the volumetry measure).

Supervised regression was performed using multilayer feedforward artificial neural networks. Multilayer perceptrons are universal approximators: when good care is taken to control their complexity, they can provide better fitting than classical polynomial regressions (Haykin, 2009). We used a 2-layer perceptron, with a non-linear (sigmoid) hidden layer and a linear output. The network inputs were the two best features selected using OFR. Regression was performed using a second order gradient descent approach, with the Levenberg–Marquart algorithm (Pujol, 2007). Performances were estimated using a leave-one-out approach:

(I) One sample was taken out of the database.
(II) The network was then trained on the remaining samples, and afterwards tested on the excluded sample.
(III) The same estimation was performed iteratively for all samples of the database. The overall classification of the excluded samples is the leave-one-out error, which is a good estimate of the generalization error.

We tuned the network complexity by manipulating the number of hidden units (from 0 to 5), according to the leave-one-out error (Dreyfus, 2005).

Data analyses were performed using Matlab 2013a (Mathworks^®^).

## Results

### Behavioral Results

We performed independent sample t-tests on all the measures of interest. We found significant differences for all measures in favor of better performance in young adults than in older adults, except for FLU [*t*(25) = 1.71, *p* = 0.1]: EAM [*t*(25) = 7.56, *p* < 0.001], EPI [*t*(25) = 3.34, *p* < 0.01], TMTB-A [*t*(25) = 4.24, *p* < 0.001], INHIB [*t*(25) = 6.72, *p* < 0.001], UP-D [*t*(25) = 2.41, *p* < 0.05], VSS [*t*(25) = 4.57, *p* < 0.001], and STB [*t*(25) = 3.07, *p* < 0.01].

### fMRI Results

#### Clustering

**FIGURE 1:**
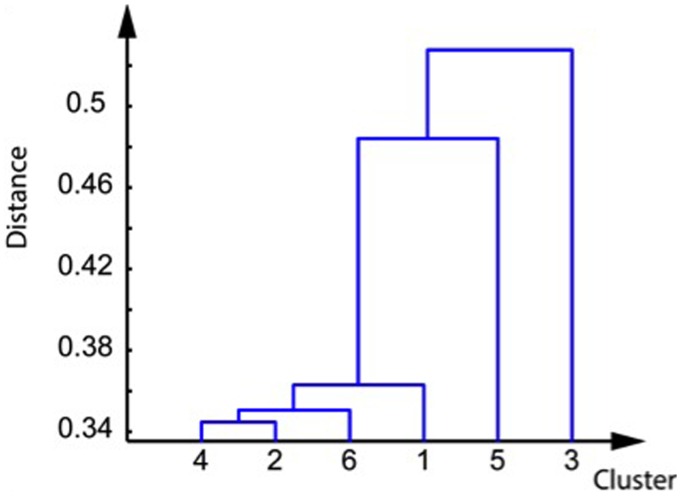
**Dendrogram of the clusters, computed from their cosine distance matrices**.

The best silhouette was obtained using six clusters. The within-cluster mean of distance to centroid was below 0.25 for all clusters except for cluster 6. Cluster 1 is particularly large and contains 16 networks (**Table 1**). Using the distance matrices between the cluster elements, we extracted the cluster hierarchy (**Figure 1**), which shows three blocks in these six clusters, composed of cluster 3, cluster 5, and the remaining clusters.

**Table 1:** Clusters of pairs of networks (cosine distance measure), ordered according to their homogeneity and dimension.

clusters	1	2	3	4	5	6
homogeneity	0.18	0.18	0.18	0.20	0.21	0.35
networks	Lvattfr-dmfr	Mot-sal	Mot-dmps	Dmfr-dmps	Lvattfr-lvattps	Mot-dmfr
	Lvattfr-dmps	Mot-lvattfr	Mot-vis	Dmfr-dmtemp	Lvattfr-rvattfr	Mot-dmtemp
	Lvattfr-dmtemp	Mot-lvattps	Dmfr-vis	Dmps-dmtemp	Lvattfr-rvattps	Mot-front
	Lvattfr-front	Mot-rvattfr	Dmps-vis	Dmps-front	Lvattps-rvattfr	Sal-lvattfr
	Lvattps-dmfr	Mot-rvattps	Dmtemp-vis	Dmtemp-front	Rvattfr-rvattps	Sal-lvattps
	Lvattps-dmps	Sal-vis	Front-vis		Dmfr-front	Sal-rvattfr
	Lvattps-dmtemp	Lvattfr-vis				Sal-rvattps
	Lvattps-front	Lvattps-rvattps				Sal-dmfr
	Rvattfr-dmfr	Lvattps-vis				Sal-dmps
	Rvattfr-dmps	Rvattfr-vis				Sal-dmtemp
	Rvattfr-dmtemp					Sal-front
	Rvattfr-front					
	Rvattps-dmfr					
	Rvattps-dmps					
	Rvattps-dmtemp					
	Rvattps-front					

*Lvattfr, left ventral attentional frontal; Lvattps, left ventral attentional posterior; Rvattfr, right ventral attentional frontal; Rvattps, right ventral attentional posterior; dmfr, default mode frontal; dmps, default mode posterior; dmtemps, default mode temporal; front, frontal; Mot, motor; Sal, salience; vis, visual*.

#### Prediction of Age

**FIGURE 2:**
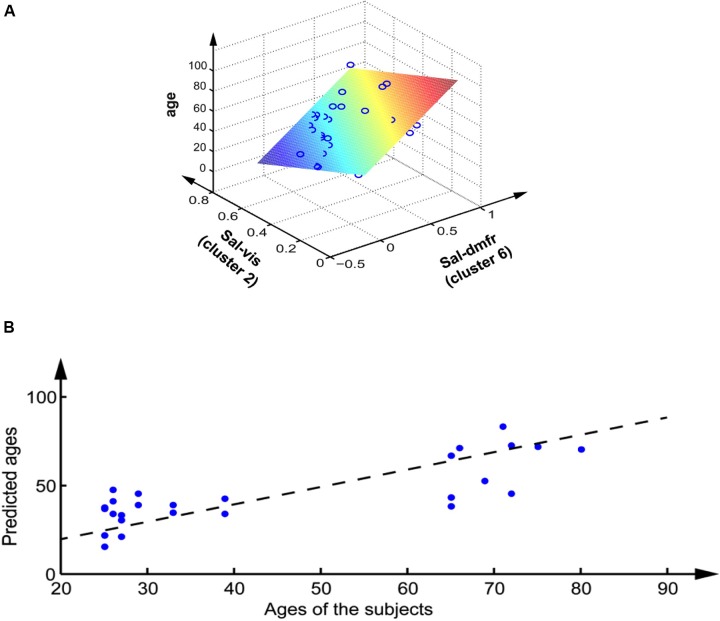
**Prediction of the age of the subjects from their rs-fMRI inter network activity. (A)** Linear regression, each circle represents a subject, the colored plane represents the linear regression. **(B)** Non-linear regression based on the multilayer perceptron, on the variables after volume correction, on the leave-one-out validation set. Each dot represents a subject; the dashed line represents the optimum.

The best two pairs of networks to predict age according to the OFR algorithm were sal-vis and sal-dmfr, corresponding to clusters 2 and 6. These two clusters belong to the same block on the dendrogram. A Pearson correlation *R*^2^ of 0.61 was obtained (*p* = 2.07^∗^10^-5^) using these two variables. The best neural network architecture selected had four hidden units. A leave-one-out mean-squared error of 0.06 was achieved for the age prediction, corresponding to a generalization error of 6.69 ± 5.3 years (the prediction error reached 9.10^-7^ on the training set) (**Figure 2**). Given the pivotal role of the salience network in predicting age and cognitive variables, we conducted *post hoc* analyses on the within network connectivity. Interestingly, we found that the connectivity between the cingulate gyrus and insula (which are the main hubs of the salience network) was reduced in the group of old adults (*t* = -2.52, *p* < 0.05). Moreover the connectivity between these two brain regions showed a negative correlation with age in the old adults (*R* = -0.7, *p* < 0.05), but not in the young adults group (*R* = -0.03, *p* = 0.9). Nevertheless, when accounting for within network connectivity, we were still able to significantly predict the age from the between network connectivity between the sal-dmnf and the sal-vis (*R* = 0.368, *p* = 4.06 E-03).

#### Prediction of Cognitive Variables

**FIGURE 3:**
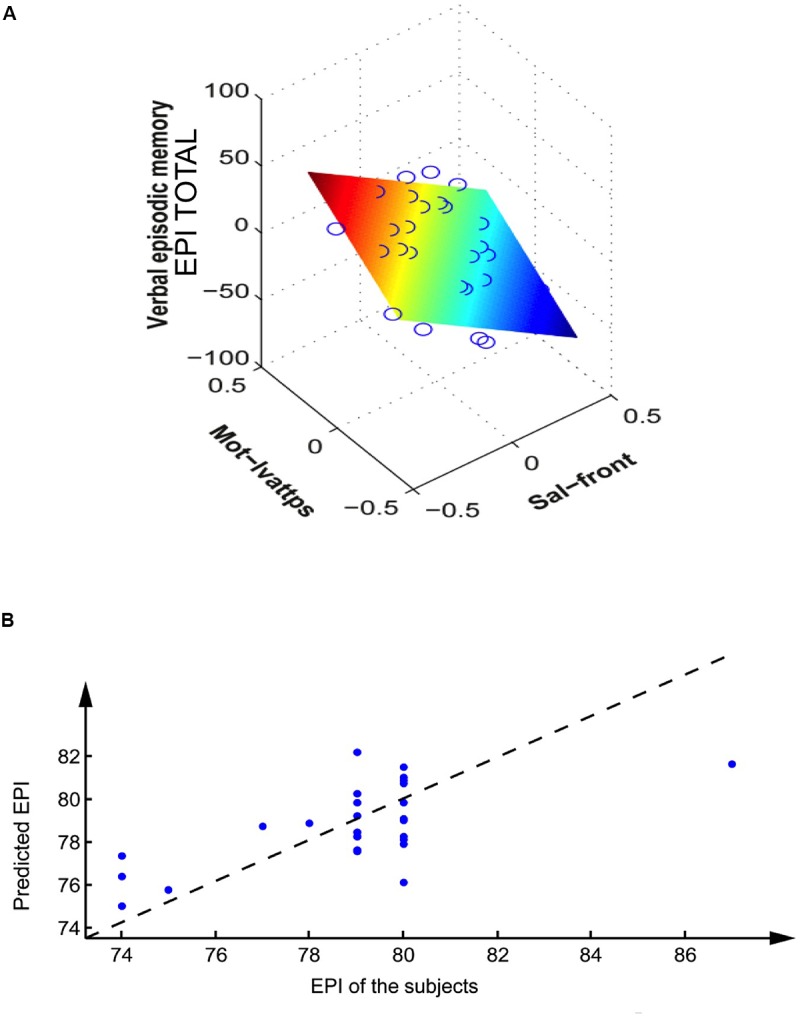
**Prediction of EPI total (verbal episodic memory) of the subjects from their rs-fMRI inter network activity. (A)** Linear regression on the corrected variables (correction by volume and age). Each circle represents a subject, the colored plane represents the linear regression. **(B)** Non-linear regression based on the multilayer perceptron, on the variables after volume and age correction, on the leave-one-out validation set. Each dot represents a subject; the dashed line represents the optimum (obtained with a linear perceptron without hidden units).

Seven cognitive variables were successfully predicted from rs-fMRI pairs of networks (see **Table 2**): EPI total, STB, EAM, FLU, TMT B-A, and VSS Scores. The best prediction was obtained with EPI total, which had the highest linear correlation p-values and a very low leave-one-out generalization error (6%). Moreover, three of these variables were regressed efficiently using a neural network, with a satisfactory leave-one-out error (below 15%). These three variables (EPI total, STB, EAM) were predicted from the same clusters (clusters 6 and 2), which are the same as the age predicting clusters. The four remaining variables had poorer leave-one-out errors (above 20%). Three variables (FLU, TMT B-A, VSS) had cluster 5 in common. Within those three, the last two variables (TMT B-A, and VSS) shared the exact same networks (belonging to cluster 5 and cluster 6). Two other variables showed the same network (cluster 6), but did not show significant correlations after age correction (*p* > 0.05): INHIB score, UP-D score (**Table 2**; **Figure 3**).

**Table 2:** Cognitive variables prediction from fMRI pairs of networks.

Variable	Networks	Clusters	Significance	Corrected significance	Learning error
EPI total	Sal-front	6, 2	*R*^2^ 0.59	*R*^2^ 0.46	0.06
	Mot-lvattps		*p* = 3.1^∗^10-5	*p* = 7.9^∗^10-4	
STB	Sal-dmps	6, 2	*R*^2^ 0.31	*R*^2^ 0.44	0.14
	Rvattps-vis		*p* = 1.3^∗^10-2	*p* = 1.2^∗^10-3	
EAM	Sal-dmtemp	6, 2	*R*^2^ 0.60	*R*^2^ 0.30	0.14
	Sal-vis		*p* = 2.4^∗^10-5	*p* = 0.015	
FLU	Rvattfr-dmfr	1, 5	*R*^2^ 0.44	*R*^2^ 0.35	0.21
	Lvattps-Rvattfr		*p* = 1.3^∗^10-3	*p* = 6.6^∗^10-3	
TMT B-A	Lvattps-Rvattfr	5, 6	*R*^2^ 0.49	*R*^2^ 0.38	0.21
	Sal-rvattps		*p* = 4.4^∗^10-4	*p* = 3.9^∗^10-3	
VSS	Lvattps-Rvattfr	5, 6	*R*^2^ 0.38	*R*^2^ 0.33	0.29
	Sal-rvattps		*p* = 4.3^∗^10-3	*p* = 9.9^∗^10-3	
INHIB	Sal-front	6	*R*^2^ 0.56	*R*^2^ 0.22	0.11
			*p* = 8.9^∗^10-5	*p* = 0.053	
UP-D	Sal-dmtmp	6	*R*^2^ 0.34	*R*^2^ 0.16	0.19
	Sal-rvattps		*p* = 8.3^∗^10-3	*p* = 0.14	

*The network column indicates the two best pairs of networks selected using OFR algorithm. The cluster column indicates the corresponding clusters. The significance column indicates Pearson correlation results using the selected networks. Corrected significance is the result of Pearson correlation analysis after correction by the age variable. Learning error is the leave-one-out generalization error on the normalized output. Grey color indicated satisfactory leave-one-out error (below 15%). EPI total, total score episodic memory; STB, short term binding; EAM, episodic autobiographical memory; FLU, verbal fluency; TMTB-A, difference of time execution between the part B and the part A; VSS, visuo-spatial span; INHIB, difference between the time of denomination of the interference part and the denomination part; UP-D, updating in working memory.*

**FIGURE 4:**
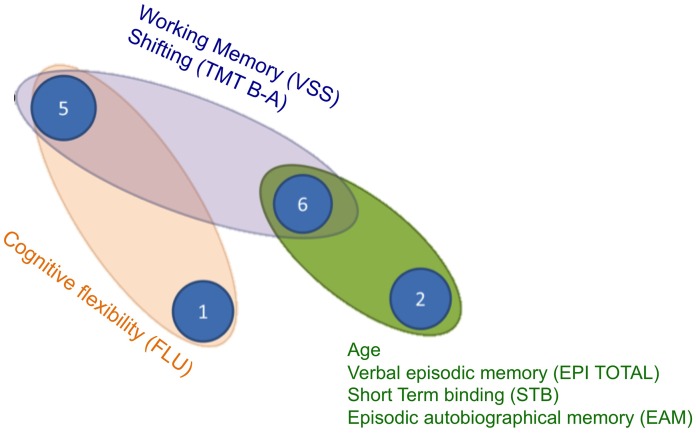
**Cognitive network of clusters.** The distances between each cluster represents the dendrogram distance already illustrated in **Figure 1**. Each ellipse indicates groups of variables successfully predicted by pairs of the same activation networks. EPI total, total score verbal episodic memory; STB, short term binding; EAM, episodic autobiographical memory; FLU, verbal fluency; TMTB-A, difference of time execution between the part B and the part A; VSS, visuo-spatial span.

Furthermore by combining the measures of distances and the variable predictions, it was possible to draw a general graph of the relationships between the cognitive activation clusters and the predicted variables independently of age (**Figure 4**). On that graph, we can identify three functional blocks (clusters 2 + 6; clusters 6 + 5, clusters 5 + 1). One can see the central importance of cluster 6, which is involved in all but one variable prediction (verbal fluency FLU) and was identified as a general cluster of cognitive decline: this cluster also predicts age, and in addition most cognitive variables when excluding age effects. The functional block of verbal fluency FLU is the only one that is not related to cluster 6. Cluster number 4 was not associated directly to any of the investigated variables (but as it is close to clusters 2 and 6, it could nevertheless be used as a replacement cluster for the prediction of the variables Age, EPI total, STB, and EAM).

## Discussion

In this work, we showed that the connectivity profile between specific RSN networks predicts both the age of the subjects and their cognitive abilities. The originality of the study consisted in using a machine-learning approach to predict age and cognitive performance from the functional connectivity patterns characterized in the brain. In particular we reported that the connectivity between the salience and visual networks, and the salience and the anterior part of the DMN, were the best features in predicting the age of the subjects. Moreover, connectivity between the salience and different specific networks predicted the episodic performance (Sal-front), the short-term binding (Sal-dmps) and the episodic autobiographical score (Sal-dmtemp, Sal-vis), independently of the age of the subject. Finally, the connectivity between the salience and the frontal networks predicted inhibition and updating performance, but this correlation was no longer significant after removing the effect of age.

Our findings suggest a pivotal role of the salience network in the neural reorganization in aging. The connectivity profile of this network was not only the best feature to predict age, but was also involved in the prediction of several cognitive functions, such as verbal episodic memory, short-term binding and episodic autobiographical memory. Nevertheless, these scores were also predicted independently of the age of the subject, thus the variability in the strength of connectivity between these networks seems more linked to the variability in cognitive functions *per se* than to the effect of aging. On the contrary, the prediction of inhibitory and updating performances dropped when age was taken into account, suggesting that the connectivity profile of the network predicting inhibition and updating in working memory is particularly sensitive to the effect of aging. These findings are in line with studies that have related cognitive deficits in the elderly to a reduction in inhibitory control (Hasher et al., 1999). The central role of the salience network reported here is coherent with recent findings showing that the connectivity profile of this network was one of the best predictors of age (Meier et al., 2012), and that the connectivity between the salience and the visual networks and the salience and the temporal networks was correlated with age (Onoda et al., 2012). In accordance with the latter study we showed that one of the best features predicting age was the connectivity between the salience and the visual network. On the contrary, while Onoda et al. (2012) did not report robust alteration of the default mode with age, we found that another feature involved in age prediction was the connectivity between the salience and the anterior portion of the default mode. The salience network, composed of the anterior cingulate cortex (ACC) and the insula, is thought to code behaviorally relevant information (Seeley et al., 2007). One recent proposal is that this network, in particular the insular cortex, may promote the dynamic switch between other large scale networks (e.g., the default mode and the central executive network) in order to ensure adaptive behavior *via* flexible cognitive control mechanisms (Sridharan et al., 2008; Menon and Uddin, 2010). Recent studies have reported an altered salience network in normal aging. In particular He et al. (2014) showed that functional and structural impairment of the salience network may occur early in normal aging and that functional disconnection between this network and the central executive network and the DMN may also be associated with normal aging and Alzheimer’s disease.

Moreover, the connectivity between the salience network and specific networks predicted different cognitive functions. In particular, the connectivity with the frontal networks predicted episodic memory performance. This finding is in line with the role of the frontal cortex in both encoding and retrieval of episodic memory (e.g., Spaniol et al., 2009). Concerning episodic autobiographical memory, we reported that performance was predicted by the connectivity between the salience network and the temporal component of the DMN, comprising the hippocampus. The role of the hippocampus in episodic autobiographical memory is well established (see for example two meta-analyses: Svoboda et al., 2006; Martinelli et al., 2013c). A recent investigation by Grady et al. (2015) pointed out that the salience network is also engaged during recall failures. In particular these findings suggest that the dedifferentiation of functional connectivity within the salience network across memory conditions and the reduction in functional coupling between it and the PFC may indicate weak inter-network communication either while retrieval is attempted or when monitoring takes place after retrieval has failed.

In addition, supplementary results showed that the connectivity between crucial hubs of the salience network, such as cingulate gyrus and insula, was reduced in elderly subjects and that the connectivity between these two regions showed a negative correlation with age only in the old adults group. These findings highlight the involvement of the principal hubs of the Salience Network in neurocognitive aging.

Moreover, this reduction of connectivity correlated with age only in the elderly group. Nevertheless, we were still able to predict the age of the subjects from between networks connectivity when within network connectivity was taken into account. These findings suggest that while age is accompanied by an alteration of the intrinsic dynamic of the salience network; inter networks connectivity seems to represent more robust predictors of age.

Finally, the connectivity between the posterior portion of the DMN, comprising the temporo-parietal junction (TPJ) and the precuneus/posterior cingulate cortex, and the salience network predicted short-term binding in working memory. The temporo-parietal junction, beyond attentional and social functions (Scholz et al., 2009), has been linked to working memory processes (Anticevic et al., 2010). Moreover, interestingly, a recent study reported a direct involvement of the TPJ in visual feature binding (Pollmann et al., 2014). Thus, the role of this structure seems coherent with the cognitive demands of our short-term binding task. Taken together these findings suggest that the salience network allocates the necessary cortical resources to other networks that are specialized in the task at hand. Moreover, the link between the connectivity of these networks and the corresponding cognitive functions does not seem to be particularly sensitive to aging, since correlations remain significant even after the effect of age is taken into account.

On the contrary, the correlation between the connectivity of the salience and the frontal network and inhibition performance was affected by age. The link between both the ACC, one of the nodes of the salience network, and the PFC and inhibition, especially during the Stroop task, is well documented (Laird et al., 2005; Nee et al., 2007). Moreover, a recent study showed that performance on the Stroop task was associated with the integrity of fiber tracts connecting these structures in aging, even when controlling for general processing speed (Wolf et al., 2014). Interestingly another recent study has shown that the role of the salience network changes over the life span, which may have implications for the early detection of pathophysiology in elderly populations (Archer et al., 2016).

## Conclusion

The present study highlights the crucial role of the salience network in cognitive aging related to specific cognitive decline in particular in episodic memory and executive functions. This network is situated at the interface of the cognitive, motivational and affective system of the human brain. It plays a crucial role in identifying the most biologically and cognitively relevant endogenous and external stimuli in order to adaptively guide behavior (Menon, 2015). Indeed it can be considered as a key brain system for integrating cognition, action and feelings.

Further research on normal aging and pathological populations is needed to better characterize the role of disrupted connectivity in the preclinical phase of neurodegenerative disease. Within this context the early detection of functional connectivity abnormalities may be helpful for early diagnosis of the diseases with the aim of characterizing a pathological signature of the reorganization of brain networks in pathological aging.

## Author Contributions

VLC, MS, FV, and PP wrote the article. MS and SL did the neuroimaging exams. CM, FV did the data processing and data analyses. PP, TG, and CO conceptualized the experiment. All the authors contributed to the final draft of the article.

## Conflict of Interest Statement

The authors declare that the research was conducted in the absence of any commercial or financial relationships that could be construed as a potential conflict of interest.

## Footnotes

1 http://www.nitrc.org/frs/shownotes.php?release_id=916

2 www.fil.ion.ucl.ac.uk/spm
