# Supplementary material for: Cognitive Decline and Reorganization of Functional Connectivity in Healthy Aging: The Pivotal Role of the Salience Network in the Prediction of Age and Cognitive Performances
Source: Front Aging Neurosci. 2016 Aug 29;8:204. doi: 10.3389/fnagi.2016.00204 (PMC5003020; doi:10.3389/fnagi.2016.00204)
Supplement: Supplementary file 1 [file Table_1.DOCX]

**Appendix 1. List of networks and corresponding brain regions**

| **Network** | **Label** |
| --- | --- |
| Lvattfr | L_Frontal_Mid |
|  | L_Frontal_Mid_Orb |
|  | L_Precentral |
| Rvattfr | R_Frontal_Inf_Tri |
|  | R_Precentral |
| Lvattps | L_Cerebellum |
|  | L_Parietal_Inf |
|  | L_Precuneus |
| Rvattps | R_Cerebellum |
|  | R_Temporal_Inf |
|  | R_Angular |
|  | R_Parietal_Inf |
| dmfr | L_Frontal_Mid_Orb |
|  | L_Frontal_Sup_Medial |
|  | L_Frontal_Sup |
|  | R_Frontal_Sup |
|  | L_Frontal_Medial |
|  | R_Frontal_Medial |
| dmps | L_Cerebellum |
|  | R_Cerebellum |
|  | L_Temporal_Mid |
|  | R_Temporal_Mid |
|  | L_Hippocampus |
|  | R_Hippocampus |
|  | L_ParaHippocampal |
|  | R_ParaHippocampal |
| dmtemps | L_Precuneus |
|  | L_Cerebellum |
|  | L_Angular |
|  | R_Angular |
| Front | L_Frontal_Mid |
|  | R_Frontal_Mid |
|  | L_Cingulum_Mid |
| Sal | L_Insula |
|  | L_Temporal_Pole_Sup |
|  | L_Cingulum_Mid |
| Vis | L_Calcarine |
|  | L_Lingual |
|  | R_Lingual |
|  | L_Cuneus |
|  | L_Fusiform |
|  | L_Occipital_Sup |
| Mot | L_Temporal_Sup |
|  | L_Postcentral |
|  | R_Postcentral |
|  | L_Heschl |
|  | L_Rolandic_Oper |
|  | L_Supp_Motor_Area |

L=left, R=right, Sup=superior, Mid=middle, Inf=inferior, Orb=orbital, Tri=triangular, Oper=operculum

Lvattfr=Left ventral attentional frontal, Lvattps= Left ventral attentional posterior, Rvattfr= Right ventral attentional frontal, Rvattps= Right ventral attentional posterior, dmfr=default mode frontal, dmps= default mode posterior, dmtemps=default mode temporal, Front=frontal, Mot=motor, Sal=salience, vis=visual
